# Nephrotoxicity caused by colistin use in ICU: a single centre experience

**DOI:** 10.1186/s12882-023-03334-8

**Published:** 2023-10-13

**Authors:** Isa Kilic, Yavuz Ayar, İlkay Ceylan, Pınar Kucukdemirci Kaya, Gulbahar Caliskan

**Affiliations:** 1 Department of Anesthesiology and Intensive Care, Ministry of Health, Bursa City Hospital , Bursa, Turkey; 2 Department of Nephrology and Internal Medicine, Health Sciences University, Bursa City Hospital, Bursa, Turkey; 3 Department of Anesthesiology and Intensive Care, Ministry of Health, Yuksek Ihtisas Training and Research Hospital, Bursa, Turkey; 4 Department of Anesthesiology and Intensive Care, Bursa Uludag University, Faculty of Medicine, Bursa, Turkey

**Keywords:** Intensive care unit, Colistin, Acute kidney injury, Vasopressor

## Abstract

**Background:**

We aimed to determine the risk factors that may be associated with colistin-induced acute kidney injury (AKI) to promote the safer use of colistin in the treatment of nosocomial infections caused by multidrug-resistant Gram-negative bacteria in intensive care units.

**Materials and methods:**

This retrospective observational study was conducted among adult patients who received a minimum of 48 h of intravenous colistin from January 2020 to December 2020 at the intensive care unit of a tertiary care hospital. AKI diagnosis and staging were made based on the Kidney Disease Improving Global Outcome Criteria.

**Results:**

Of 148 patients who received intravenous colistin at a daily dose of 9 million IU, 54 (36%) developed AKI. In the univariate analysis, age, Charlson comorbidity index, APACHE II score, duration of colistin treatment, basal creatinine level, use of vasopressors, and vancomycin were significantly associated with AKI (p < 0.05). The multivariate analysis revealed that the independent predictor of AKI was the use of vasopressors (OR: 3.14; 95% confidence interval: 1.39–97.07; p = 0.06).

**Conclusion:**

The use of vasopressors in critically ill patients was independently associated with AKI developing during colistin treatment.

## Introduction

Nosocomial infections caused by multidrug-resistant Gram-negative bacteria continue to threaten life in intensive care units (ICU). Increasing drug resistance often makes it inevitable to add colistin to the treatment of these infections [1–3]. The most important side effect that necessitates a dose limitation in colistin use is colistin-induced acute kidney injury (AKI). Previous studies have reported that the rate of AKI due to colistin can reach up to 70% [4–6]. Nephrotoxicity increases hospital and ICU mortality [7]. Therefore, identifying and correcting modifiable risk factors for colistin-induced AKI will contribute to reducing mortality.

In this study, we aimed to determine the risk factors that may be associated with colistin-induced AKI to promote the safer use of colistin in the treatment of nosocomial infections caused by multidrug-resistant Gram-negative bacteria in the ICU.

## Materials and methods

### Setting

After approval from the hospital ethics committee (approval number 2021-17/2), a retrospective cohort study was conducted in the 51-bed ICU of a tertiary care hospital (Bursa City Hospital) in Bursa, Turkey. Patients who received intravenous colistin between 1 and 2020 and 31 December 2020 were included in the study.

### Patients, study design, and data collection

ICU patients over 18 years of age, with an estimated glomerular filtration rate (eGFR) of at least 60 mL/min/1.73 m^2^, calculated using the Chronic Kidney Disease Epidemiology Collaboration equation, who had received colistin therapy for at least 48 h and were included in the study. In our study, we excluded patients with known kidney diseases at baseline, including those with eGFR greater than 60 mL/min/1.73 m² as calculated by the CKD-EPI equation, but with evidence of albuminuria. Additionally, individuals with urine sediment abnormalities, electrolyte and other abnormalities indicative of tubular disorders, any abnormalities detected through histological examination, structural abnormalities identified through imaging modalities, and patients with a history of kidney transplantation were also excluded from the study. For patients receiving multiple courses of colistin, only the first treatment was considered. Colistin was started according to the culture results and/or the decision of the infectious diseases specialist. A loading dose of 9 million international units (MIU) intravenous (IV) was administered, followed by a maintenance dose of 4.5 MUI/day IV twice daily 12 h later, as recommended by our hospital protocols. In patients who developed AKI, we administered colistin doses according to the International Consensus Guidelines for the Optimal Use of the Polymyxins [8].

The study data were obtained from the electronic health records of our hospital. Age, gender, length of stay (LOS) ICU, LOS hospital, Acute Physiology and Chronic Health Evaluation II (APACHE II) score (at colistin initiation), underlying diseases, Charlson comorbidity index (CCI), and duration of colistin therapy were recorded. Concomitant use of other nephrotoxins (nephrotoxic antimicrobial agents, nonsteroidal anti-inflammatory drugs [NSAID]), causative organisms, and site of infection were recorded. During the study, the type of replacement was recorded for patients who needed renal replacement.

### Definitions

The eGFR value was calculated with the Chronic Kidney Disease Epidemiology.

Collaboration formula. AKI was defined by applying the Kidney Disease Improving Global Outcome Criteria (KDIGO) recommendations within 48 h or 7 days from the initiation of colistin treatment. Specifically, AKI was defined as a ≥ 0.3 mg/dL increase in serum creatinine (SCr) from baseline at 48 h, or a 1.5× increase within 7 days [9]. Basal serum creatinine levels were used to define AKI according to the KDIGO criteria. We defined basal serum creatinine as the lowest creatinine value if the patient’s previous creatinine values were known, and the serum creatinine level on the first day of intravenous colistin administration if the previous creatinine values were not known. Since daily urine output data were not sufficient for all patients, the urine amount criterion was not used in the diagnosis and staging of AKI. Health care-associated pneumonia, urinary tract infection, central nervous system infection, intra-abdominal infection, bloodstream infection, and wound infection (surgical wound infection and the others) were defined according to the 2008 standardised criteria of the Center for Disease Control and Prevention [10].

Hypertension is defined as a condition in which individuals either use antihypertensive medication or have blood pressure readings consistently above 140/90 mmHg. Patients previously diagnosed with chronic obstructive pulmonary disease (COPD) by a pulmonologist and under follow-up were classified as having COPD. Patients with a history of coronary stenting or coronary bypass surgery were classified as having coronary artery disease (CAD). Patients with a previous diagnosis of diabetes (fasting plasma glucose > 126 mg/dl measured on at least two separate days) were considered as having diabetes mellitus (DM). Malignancy was defined as patients with malignant disease diagnosed by biopsy. Patients diagnosed with heart failure by a cardiologist and receiving heart failure treatment were classified as congestive heart failure (CHF). Cerebrovascular disease (CVD) was defined as stroke, transient ischaemic attack (TIA), aneurysm, and cerebral vascular malformation.

### Statistical analysis

Data analysis was performed using the Statistics Package for Social Science (SPSS 23.0-IBM, NY, USA). A two-tailed Kolmogorov–Smirnov test was applied to examine whether the continuous quantitative variables followed a Gaussian distribution. Characteristics of patients, that is, *n* (percent) or median (minimum-maximum) for categorical and continuous variables, respectively, were compared among groups using chi-square or Mann–Whitney tests, as appropriate. Logistic regression analysis was performed to determine the risk factors affecting the development of nephrotoxicity. Factors that were statistically significant in the univariate model were included in the multivariate model. The *p* value was set at < 0.05 for statistical significance.

## Results

**Fig. 1:**
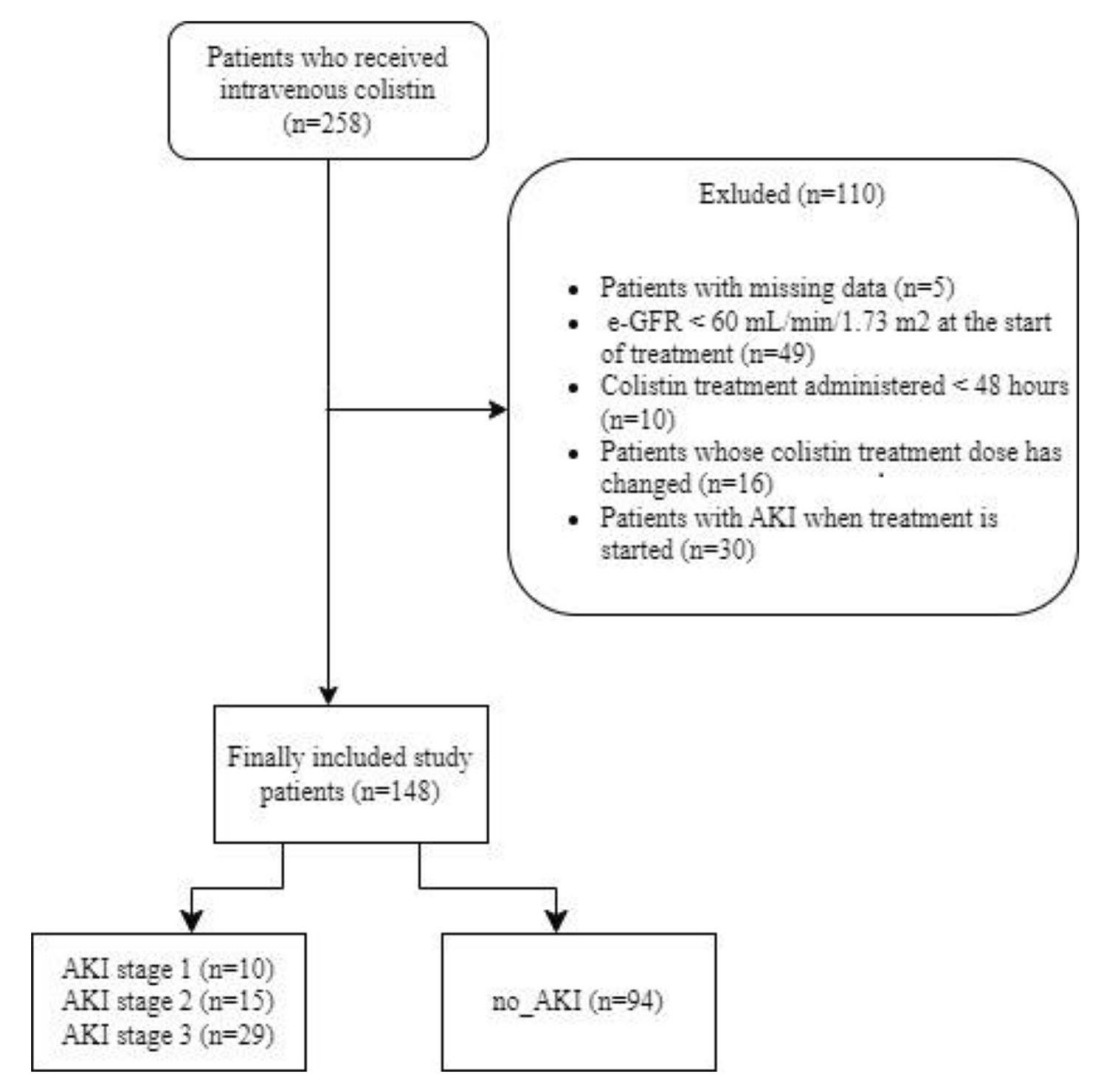
Flow diagram of study screening. AKI: Acute Kidney Injury

Of the 258 retrospectively screened patients for IV colistin use in the ICU, 148 were eligible for inclusion in the study (Fig. 1). Within the scope of the study, 148 patients, including 25.7% (38) women and 74.3% (110) men, were analysed. The age of the patients ranged from 21 to 91 years, and the median age was 65 years. Their median length of hospitalisation was 41 (110–214) days. The median duration of colistin treatment was 12 (3–36) days. The number of patients who received vasopressors was 60 (40.5%). The distribution of demographic and clinical findings according to the developmental status of AKI in the participants is provided in Table 1. As shown in the table, there was a statistically significant difference between the two groups in terms of age, APACHE II score, duration of colistin treatment, values of baseline creatinine, use of vasopressor, and mortality (p < 0.05). Age, APACHE II, duration of colistin treatment, creatinine levels, and use of vasopressor were found to be higher in patients who developed AKI compared to the no-AKI group. In total, 65% (39 patients) of vasopressor users died, compared to 21.6% (19 patients) of non-users (p < 0.001). Colistin was administered to 136 of the 148 patients included in the study according to the culture results; however, it was empirically administered in 12 patients.

**Table 1:** Distribution of Demographic and Clinical Findings of the Patients

Characteristics	Total (n = 148)	Non-AKI (n = 94)	AKI (n = 54)	p-value
Gender, n (%)				1.000
Female	38 (25.7)	24 (25.5)	14 (25.9)	
Male	110 (74.3)	70 (74.5)	40 (74.1)	
Age, year, median (min-max)	65 (21–91)	61 (21–89)	70.5 (40–91)	< 0.001
APACHE II score (when colistin therapy is started) (min-max)	17 (2–49)	16 (2–43)	20.5 (5–49)	0.004
ICU admission diagnosis n (%)				
Pneumonia	135 (91.2)	86 (91.5)	49 (90.7)	1.000
Bloodstream infection	11 (7.4)	6 (6.4)	5 (9.3)	0.530
Urinary tract infection	6 (4.1)	5 (5.3)	1 (1.9)	0.416
Soft tissue infection	4 (2.7)	1 (1.1)	3 (5.6)	0.138
ICU admission CCI score, median (min-max)	3 (0–10)	3 (0–10)	4 (0–9)	0.002
**Comorbidity, n (%)**	123 (83.1)	75 (79.8)	48 (88.9)	0.232
HT	37 (25)	21 (22.3)	16 (29.6)	0.430
DM	29 (19.6)	19 (20.2)	10 (18.5)	0.972
CHF	31 (20.9)	15 (16)	16 (29.6)	0.079
COPD	24 (16.2)	18 (19.1)	6 (11.1)	0.296
CVD	21 (14.2)	11 (11.7)	10 (18.5)	0.368
Malignancy	31 (20.9)	19 (20.2)	12 (22.2)	0.937
CAD	36 (24.3)	20 (21.3)	16 (29.6)	0.347
ICU LOS, days, median (min-max)	32 (3–178)	32 (3–178)	32 (4–128)	0.484
Hospital LOS, days, median (min-max)	41 (10–214)	46 (11–214)	39.5 (10–198)	0.873
From onset of colistin to AKI development time, days, median (min-max)	6 (1–29)	NA	6 (1–29)	NA
Duration of colistin treatment, days, median (min-max)	12 (3–36)	11.5 (3–30)	13.5 (3–36)	0.045
Duration of colistin treatment > 14 days, n (%)	52 (35.1)	29 (30.9)	23 (42.6)	0.207
Basal creatinine level, mg/dl, median (min-max)	0.6 (0.2–1.2)	0.6 (0.2–1.1)	0.7 (0.2–1.2)	0.027
KDIGO, n (%)				NA
Stage-I	10 (18.5)	NA	10 (18.5)	
Stage-II	15 (27.8)	NA	15 (27.8)	
Stage-III	29 (53.7)	NA	29 (53.7)	
Haemodialysis, n (%)				
IHD	7 (4.7)	NA	6 (4)	
CRRT				
Vasopressor use, n (%)	60 (40.5)	28 (29.8)	32 (59.3)	< 0.001
Mortality, n (%)	58 (39.2)	28 (29.8)	30 (55.6)	0.004

APACHE II: Acute Physiology and Chronic Health Evaluation II, ICU: Intensive care unit, LOS: length of stay, CCI: Charlson comorbidity index, HT: hypertension, DM: Diabetes mellitus, CHF:Congestive heart failure, COPD: Chronic obstructive pulmonary disease, CVD: Serebro vascular disease, CAD: Coronary artery disease, AKI: Acute kidney injury; KDIGO: Kidney Disease Improving Global Outcomes, IHD: Intermittent haemodialysis, CRRT: Continuous renal replacement therapy

Table 2 shows the distribution of the causative organism, the use of nephrotoxic agents, and growth in cultures from patients according to the developmental status of AKI in the participants. We observed a statistically significant relationship with the use of only vancomycin, one of the nephrotoxic agents (p < 0.05). The results of the logistic regression analysis, which examined the risk factors affecting the development of AKI in patients within the scope of the study, are provided in Table 3. Age, CCI, APACHE II score, duration of colistin treatment, serum creatinine at baseline, use of vasopressor, and use of vacomycin were associated with the development of AKI in the ICU at univariate analysis.

**Table 2:** Distribution of Growth in Culture and Use of Nephrotoxic Agents by Patients

Characteristics	Total (n = 148)	Non-AKI (n = 94)	AKI (n = 54)	p-value
Growths				
Blood	15 (10.1)	11 (11.7)	4 (7.4)	0.582
Urine	7 (4.7)	6 (6.4)	1 (1.9)	0.423
Wound	4 (2.7)	1 (1.1)	3 (5.6)	0.138
Causative organism, n (%)	Growth = 147	Growth = 90	Growth = 57	
*Acinetobacter baumannii*	118 (80.3)	74 (82.2)	44 (77.2)	0.593
*Klebsiella pneumoniae*	19 (12.9)	9 (10.0)	10 (17.5)	0.282
*Pseudomonas aeruginosa*	10 (6.8)	7 (7.8)	3 (5.3)	0.741
Nephrotoxic agent, n (%)	68 (45.9)	38 (40.4)	30 (55.6)	0.108
Concomitant vancomycin	51 (34.5)	26 (27.7)	25 (46.3)	0.034
Concomitant aminoglycoside	19 (12.8)	14 (14.9)	5 (9.3)	0.465
Concomitant NSAIDs	1 (0.7)	1 (1.1)	0 (0)	1.000
Concomitant amphotericin	5 (3.4)	3 (3.2)	2 (3.7)	1.000

NSAIDs: Non-steroidal anti-inflammatory drugs

**Table 3:** Analysis of Risk Factors Affecting the Development of Nephrotoxicity

Variables	Univariate		Multivariate	
	OR (95% CI)	p-value	OR (95% CI)	p-value
Age	1.04 (1.02–1.07)	0.001	1.04 (1.01–1.07)	0.070
Gender				
Female	Reference	–		
Male	0.98 (0.46–2.11)	0.958		
CCI	1.28 (1.08–1.52)	0.004	1.08 (0.85–1.37)	0.553
APACHE II score	1.05 (1.02–1.09)	0.003	1.02 (0.98–1.07)	0.277
Diagnosis ICU admission				
Pneumonia	0.90 (0.28–2.94)	0.877		
Bloodstream infection	1.50 (0.43–5.16)	0.523		
Urinary tract infection	0.34 (0.04–2.95)	0.325		
Soft tissue infection	5.47 (0.56–53.96)	0.146		
Nephrotoxic agent				
Concomitant vancomycin	2.26 (1.12–4.54)	0.023	2.05 (0.90–4.66)	0.087
Concomitant aminoglycoside	0.58 (0.20–1.72)	0.328		
Concomitant amphotericin	1.17 (0.19–7.21)	0.868		
Vasopressors using	3.43 (1.70–6.91)	< 0.001	2.80 (1.29–6.10)	0.010
Basal creatinine level	4.81 (1.22–18.99)	0.025	2.67 (0.58–12.44)	0.209
Duration of colistin treatment	1.06 (1.01–1.11)	0.046	1.04 (0.98–1.12)	0.202

CCI: Charlson comorbidity index, APACHE II: Acute Physiology and Chronic Health Evaluation II, ICU: Intensive care unit

When the variables that were found to be significant in the univariate model were re-evaluated in the multivariate model, the use of vasopressors was found to be statistically significant (p < 0.05). In the multivariate model, the use of vasopressors resulted in a 3.1 times increase in the development of AKI.

## Discussion

Nephrotoxicity is the most critical side effect requiring dose limitation when using colistin. Colistin cause cell death by binding to the lipopolysaccharide in the cell wall of gram-negative bacteria and increasing the cell wall permeability. The mechanism of renal toxicity is through increased permeability of the renal tubular epithelium, leading to cellular lysis and acute tubular necrosis [11]. The incidence of colistin-induced AKI in ICU studies has been found to be approximately 12.7–70% [6, 12, 13]. This wide incidence range can be attributed to the heterogeneous patient population used in the studies, the different definitions of nephrotoxicity, the use of polymyxins at wide dose ranges, the severity of the underlying disease, and other risk factors in the patients. In our study, which classified AKI according to KDIGO guidelines, we found that the rate of nephrotoxicity was 36%.

Norepinephrine is used as a first-line therapy in patients with septic shock to reduce the release of pro-inflammatory mediators, regulate blood pressure, and provide tissue and organ perfusion [14]. Norepinephrine significantly reduces oxygen pressure and perfusion of the renal medulla. Norepinephrine increases GFR. Increased GFR increases sodium delivery to the medullary tubules. As the increased sodium in the tubules is reabsorbed, medullary oxygen consumption increases, resulting in medullary hypoxia [15]. Decreased renal medullary perfusion, ischemia, or microvascular shunts in the renal tubular system contribute to the development of AKI [16, 17]. Gordon et al. compared the effects of norepinephrine and vasopressin infusions on the outcome of kidney injury in 778 patients with septic shock. In the study, vasopressin users had less progression to renal failure (20.8% vs. 39.6%,) and renal replacement requirements (17.0% vs. 37.7%) compared to norepinephrine users. In addition, mortality was lower in the vasopressin group than in the norepinephrine group [18]. In another recent study, the rate of colistin-induced AKI was quite high (50%) in patients taking vasopressors for septic shock (OR: 6.2; 95% CI: 1.83–21.01) [19]. Norepinephrine seems to increase the risk of AKI in patients with sepsis due to such negative effects on kidney function [20]. Due to both intrarenal perfusion redistribution due to sepsis and the negative effects of norepinephrine on kidney functions, renal tubules become more sensitive to colistin toxicity. However, some studies have found no relationship between nephrotoxicity and the use of vasopressors [21, 22]. These patients had serious infections requiring combinations of antibiotics and severely compromised clinical conditions at ICU admission. Thus, use of norepinephrine remained independently associated with increased odds of developing AKI conceivably because it was a marker of the severity of clinical conditions and organ dysfunction.

A longer duration of colistin treatment may increase the risk of AKI in patients [21, 23]. In our study, the duration of colistin use was higher in the AKI group than in the non-AKI group in univariate analyses. However, this effect was lost in the multivariate analysis. Lue et al., in a retrospective study investigating the efficacy and safety of polymyxins in 119 patients, showed that 11 [7–16] days of colistin treatment duration was an independent predictor of effective treatment in multivariate analysis but did not increase the risk of AKI [24]. This result is similar to our study in terms of the relationship between colistin treatment duration and AKI risk. The CCI score estimates the probability of dying from the disease, mostly based on the patients’ comorbidities. In the same study, the CCI score was associated with poor outcomes in the multivariate analysis (OR: 1.208, 95% CI: 1.021–1.430).

The elderly population is predisposed to AKI due to increased glomerulosclerosis, interstitial fibrosis, tubular atrophy, and decreased GFR [25]. Advanced age is an independent risk factor for AKI [26, 27]. Similar to our study, univariate analyses in an observational prospective study by Dalfino et al. showed that a high APACHE II score and advanced age were risk factors for colistin-induced AKI [12].

The most important side effect of vancomycin is AKI [28]. In a retrospective study of 249 patients treated with colistin, Shields et al. found the concomitant use of vancomycin to be an independent risk factor for AKI in a multivariate analysis. (OR = 1.98, 95% CI: 1.07–3.66) [29].

One of the limitations of our study is that it is retrospective, which cannot establish cause-effect relationship. Another limitation is that we could not fully reveal the relationship between vasopressor use and nephrotoxicity, since we did not have enough information about the exact reason for vasopressor use. The limited medical records of baseline albuminuria/proteinuria, which is associated with AKI in critically ill patients, is also a limitation of this study.

In conclusion, these patients had severe infections requiring combinations of antibiotics and severely compromised clinical conditions on admission to the ICU. Thus, the use of vasopressors remained independently associated with an increased likelihood of developing AKI, possibly because it is a marker of the severity of clinical conditions and organ dysfunction.

## Data Availability

The datasets used and/or analysed during the current study available from the corresponding author on reasonable request.
